# A cooperation experiment with white-handed gibbons (*Hylobates lar*)

**DOI:** 10.1007/s10329-023-01068-7

**Published:** 2023-05-24

**Authors:** Nora T. Kopsch, Thomas Geissmann

**Affiliations:** 1 Department of Physics, Chemistry and Biology, Linköping University, Linköping, Sweden; 2 Anthropological Department, University Zurich-Irchel, Zurich, Switzerland; 3 Department of Philosophy, Cognitive Science, Lund University, Lund, Sweden

**Keywords:** Cognition, Cooperation, Gibbons, *Hylobates*, Problem-solving, Social behavior

## Abstract

Cooperative behaviors among individuals of numerous species play a crucial role in social interactions. There is a special interest in investigating the occurrence of cooperation among apes because this knowledge could also shed light on evolutionary processes and help us understand the origin and development of cooperation in humans and primates in general. Gibbons are phylogenetically intermediate between the great apes and monkeys, and therefore represent a unique opportunity for comparisons. The aim of the present study was to discover whether or not white-handed gibbons (*Hylobates lar*) show cooperative behaviors. In order to test for the respective behaviors, the gibbons were presented with a commonly used experimental cooperative rope-pulling task. The gibbons in this study did not exhibit cooperative behaviors during the problem-solving task. However, prior training procedures could not be fully completed, hence this project constitutes only the onset of exploring cooperative behaviors in gibbons. Additional behavioral observations revealed that the gibbons spent significantly more time “out of arm’s reach to everyone”, suggesting that they are less often involved in social interactions, than other, more cooperative primates.

**Supplementary Information:**

The online version contains supplementary material available at 10.1007/s10329-023-01068-7.

## Introduction

Investigating great apes has long been of special interest because this knowledge could shed light on evolutionary processes and even help us understand the origin and development of humans, and primates more generally. Gibbons or small apes (Hylobatidae) are particularly interesting because they are the sister group to the great apes and humans, but share several primitive characteristics (e.g. ischial callosities, dagger-like upper canines) with Old World monkeys that were not retained in other apes (Geissmann 1995; Thinh et al. 2010). However, gibbons have received much less attention and been subject to much less research than their “greater” cousins (Fan and Bartlett 2017). Most gibbon studies have focused on behavior (Nicolson 1998; Parker 1973; Shepherdson et al. 1989), social structure (Brockelman et al. 1998; Fuentes 2000; Palombit 1994) and communication (Geissmann 1986, 1993; Nicolson 1998), whereas relatively little research has been directed towards their cognitive capabilities. Nevertheless, some important cognitive behaviors have already been documented. Tool-use, which is often associated with cognitive capabilities, has already been shown to exist within gibbons (Cunningham 2006; Geissmann 2009; Rumbaugh 1970). Whether gibbons are able to recognize themselves, thus exhibiting self-awareness and the onset of a theory of mind, has also been discussed. So far, studies have provided evidence both for the occurrence of self-recognition in gibbons (Heschl and Fuchsbichler 2009; Ujhelyi et al. 2000) and against it (Hyatt 1998; Inoue-Nakamura 1997; Suddendorf and Collier-Baker 2009).

Going one step further is the investigation of an individual’s possible comprehension of its own and another participant’s role in a cooperative interaction, and whether social or communicative techniques are applied to coordinate their contributions (Tomasello and Call 1997). Generally, cooperation is defined as “the behavior of two or more individuals acting together to achieve a common goal” (Boesch and Boesch 1989). The individuals are “in a situation in which neither can benefit alone, or at least not to the same degree, as when they act in concert” (Tomasello and Call 1997). Even though it was long believed that cooperative behaviors were unique to humans, several studies revealed that various other species also display cooperative behaviors (Boesch and Boesch 1989; Boesch 1994; Parish 1996).

Experiments and/or field observations have revealed cooperative behaviors across all great ape genera, including *Pan* (Boesch and Boesch 1989; Boesch 1994, 2002; Chalmeau and Gallo 1996; Hirata and Fuwa 2007; Melis et al. 2006), *Gorilla* (Sicotte 1993), and *Pongo* (Chalmeau et al. 1997; Völter et al. 2015). Numerous non-primate species have also been shown to successfully cooperate with their conspecifics, including Asian elephants (Plotnik et al. 2011), bottlenose dolphins (Eskelinen et al. 2016; Kuczaj II et al. 2015), wolves (Marshall-Pescini et al. 2017; Möslinger 2009), spotted hyaenas (Drea and Carter 2009), rooks (Seed et al. 2008), African grey parrots (Péron et al. 2011) and peach-fronted conures (Ortiz et al. 2020).

To date, it has not yet been investigated whether gibbons exhibit cooperative behaviors, and, if they do, to what extent. The only documented report on this subject emerged from Markowitz (1975, 1978). He claimed the occurrence of cooperative behaviors within a family group of captive white-handed gibbons (*Hylobates lar*). However, it is difficult to assess the relevance of his report, because no quantitative data for the occurrence of cooperative behaviors were presented. Furthermore, Markowitz’s understanding of cooperation could be challenged, since it does not quite fall within the commonly accepted and used definitions (Boesch and Boesch 1989; Tomasello and Call 1997). He merely described one gibbon manipulating the given apparatus with the result that the gibbon’s mother received the food reward. The mother, correspondingly, was never actively participating in a mutual interaction but solely profited from her son’s performances. Presumably, this kind of behavior could better be interpreted as altruistic behavior.

In this study, white-handed gibbons housed at Kolmården Wildlife Park in Sweden were presented with an experimental problem-solving task, in which two individuals were required to simultaneously pull a rope in order to receive a food reward. The aim was to provide evidence for or against the existence of cooperative behaviors in these gibbons.

## Materials and methods

### Location and time of data collection

The data collection was conducted at Kolmården Wildlife Park, situated near Norrköping town, Sweden. It took place from July 25th until December 15th, 2017.

### Animals

The animals engaged in this study were five white-handed gibbons (*Hylobates lar*), living together in a family group consisting of an adult breeding pair and their three offspring. The group composition is listed in Table 1. Young gibbons are dependent on their mother until the age of approximately 2 years (Burns and Judge 2016), and therefore, performances of the youngest offspring, Ebbot, during the cooperative problem-solving task were not included.

**Table 1:** Composition of the gibbon study group. Age classes after Geissmann (1993)

Name	Sex	Birth date	Age class at begin of study
Lelle	Male	1 Oct 1987	Adult
Elly	Female	16 Mar 1988	Adult
Elliot	Female	7 Oct 2011	Subadult
Edith	Female	22 Dec 2013	Juvenile
Ebbot	Male	30 Mar 2016	Infant

### Housing

The gibbons’ enclosure was subdivided into an indoor facility (83.6 m^2^) and an outdoor island (535 m^2^). The gibbons were free to choose between the inside and the outside area.

The animals were fed four times a day according to a semi-regular feeding schedule. Water was available ad libitum.

In order to stimulate the gibbons’ senses and their species-specific behaviors, they were provided daily with a variety of enrichment items. However, none of the gibbons had been part of a cognitive-ability-assessment study before or been trained for husbandry behaviors prior to this study. In this setting, it was not possible to divide the gibbons into different groups or “working pairs”, hence they were always together.

### Procedure and apparatus

The study was divided into three parts, two training phases and one test phase. The training phases were established to generate and develop the gibbons’ basic understanding of the physical properties and function of the test apparatus.

Two sessions per day were conducted, on five days per week, and the animals’ participation in the sessions was voluntary at all times. Positive reinforcement was used to teach the gibbons the required rope-pulling behavior.

#### Phase 1: first training phase

In the first training phase the gibbons were required to learn to pull a single rope in order to receive a food reward. The rope, hanging on the inside of the testing room, was attached to an elongated piece of plywood, called the “slide”, that would drive in through the fence when the rope was pulled, whereby the animal would access a food reward placed on the slide. A plexiglass sheet was installed on the fence to prevent the gibbons from taking the reward directly from the slide, through the fence.

To assess a gibbon’s overall performance, the ratio of correctly solved trials (i.e. pulling the rope) and failed trials (i.e. not pulling the rope) was calculated. Within a minimum of 100 trials per individual, significantly more trials had to be scored as a success than as a failure, in order to proceed with the second training phase. A trial started with baiting the station with a reward while the corresponding animal was watching. A trial ended either by pulling the rope (success), by leaving the dedicated training space (failure), or by not showing any interest over a period of 30 s (failure).

#### Phase 2: second training phase

In the second training phase the gibbons were supposed to learn that two connected rope ends were now required to be pulled simultaneously in order to get the food reward. The same apparatus was used as in the first phase, but it was partly altered, so that two ends of the rope were hanging into the testing room. The initial distances between the two rope ends were 6 cm, 11 cm, and 12 cm for Elliot, Edith, and Lelle, respectively. The variation was due to the training stations and features of the training area.

To count a gibbon’s action as correct, it had to pull both rope ends either with one hand each or both rope ends together with one hand. Using a foot instead of a hand when pulling also counted as correct. When the gibbons showed an understanding of the process, the distance between the two rope ends was progressively increased.

To assess a gibbon’s overall performance, the ratio of correctly solved trials and failed trials was calculated. A minimum of 100 trials (definition of ‘trial’ equal to the first training phase) per individual was carried out, and significantly more trials had to be scored as a success than as a failure, in order to proceed with the test phase. During the two training phases, the gibbons’ performances were documented on-site, and the time from baiting the station until the animal successfully obtained the reward was recorded to establish how fast the gibbons solved the task and if they would improve over time.

All training sessions were recorded by two cameras directed towards the testing location. The cameras used were a *GoPro Hero 4* and an *Olympus SP-610UZ*. If necessary (i.e., when two gibbons received training simultaneously), performance data was taken from the videos posterior to the training sessions.

#### Phase 3: test phase

The test used here was based on the cooperation test developed by Hirata (2003; cited in Melis et al. 2006; Hirata and Fuwa 2007). The two ends of the rope were located too far apart (149 cm) for one gibbon to work the apparatus by itself. Thus, two individuals were required to pull one end each at the same time to receive the food reward. The test phase was purely experimental, no further training was provided for the gibbons during this phase.

**Fig. 1:**
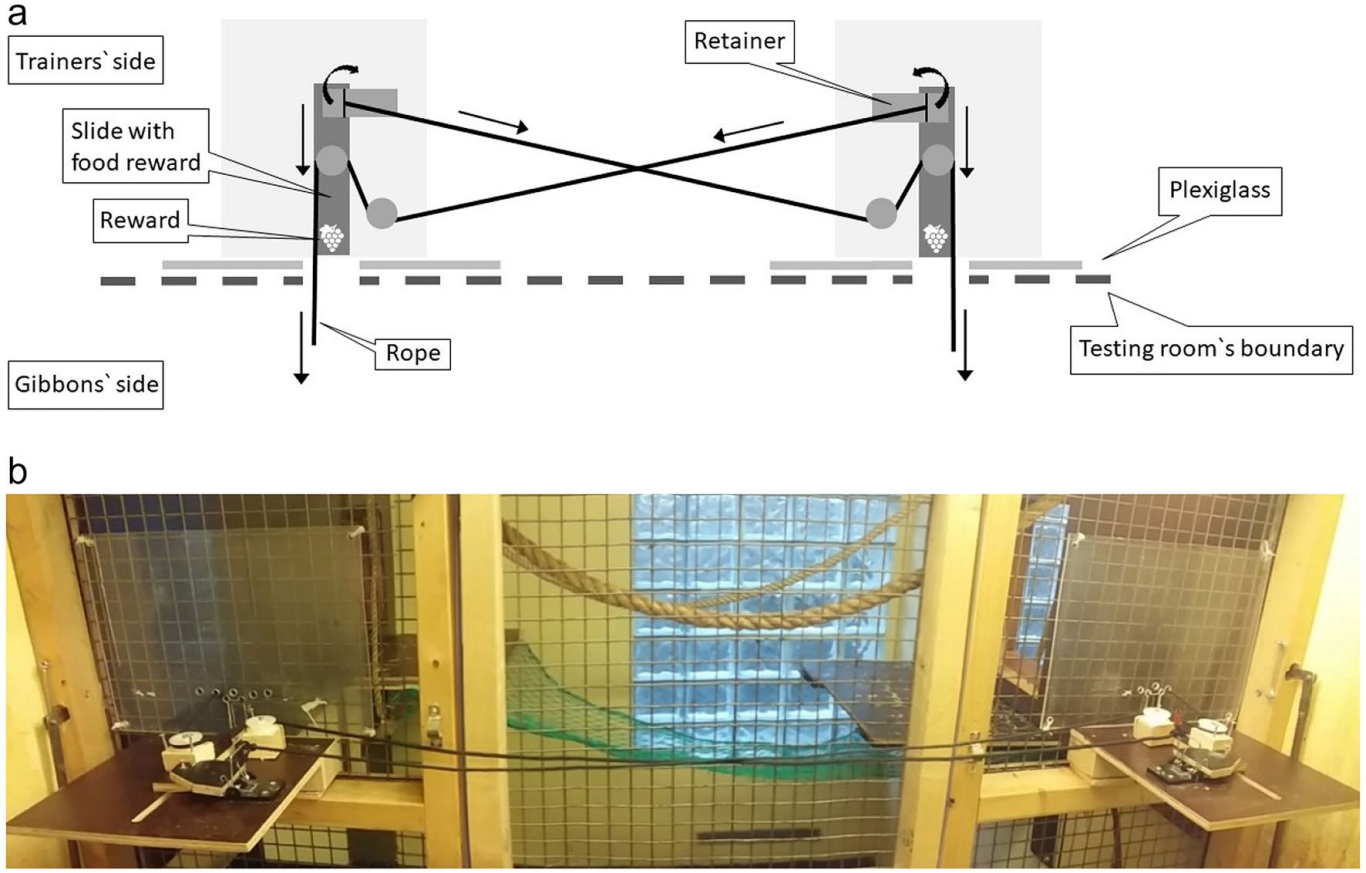
Experimental set-up for the test phase. **a** Schematic drawing of set-up from above. **b** Photograph showing the set-up from the trainer’s side

The experimental set-up for the test phase is shown in Fig. 1 (see also additional material). Two individual training stations were combined into one test apparatus that contained two single ropes, one from each. Each rope was connected to a retainer that blocked the other slide. If a gibbon pulled one rope end, the slide was not released, but the mechanism allowed to open the corresponding retainer. This enabled another gibbon to pull its slide out of the station while simultaneously unblocking the other retainer. Hence, the second slide was released as well. This mechanism ensured that two gibbons had to coordinate their actions and to work together, more specifically, when only one gibbon pulled a rope, nothing happened, and the reward was not accessible.

For the test sessions, 75-min trials were conducted. This did not only allow the gibbons to show interest in the apparatus, but also to return and try again after a while. A total of 66 test sessions was accomplished.

All test sessions were recorded by a *GoPro Hero 4*, and data was collected from the videos posterior to the test sessions. Performances of the individuals were taken into account when they effectively pulled a rope. Behaviors were rated as cooperative when two simultaneous pulls were performed by two individuals, resulting in both receiving the food reward.

#### Behavioral observations: recording of social behaviors

Alongside the test phase, social behaviors were recorded during observational sessions. Such behavioral data can aid with the interpretation of individual differences and differences between dyads during test performances, as well as assessing the gibbons’ group dynamics (e.g., strength of social bonding/ acceptance, friendly or antagonistic behaviors). Each session lasted for one hour. Two sessions per day were conducted on five days per week, resulting in a total of 66 h (i.e. 33 days) of observations. Observations were homogeneously distributed over different times of day. For the behavioral recording, scan-sampling with a one-minute-interval was applied. Behaviors were recorded according to the ethogram shown in Table 2.

**Table 2:** Ethogram used during the one-hour behavioral observations

Behavior	Descriptive term
Social grooming	Individual is investigating and cleaning the fur or skin of a conspecific
Social play	Individual is cavorting with another conspecific without displaying any obvious aggressive behaviors
Conflict	Individual displays agitated behaviors (e.g. chasing, grabbing, or hitting) towards, or in conjunction with, a conspecific
Close contact	Individual is “hugging” or “cuddling” a conspecific or is carried by a conspecific
Within arm’s reach	Individual is close enough to a conspecific to be able to grab or touch it and could be grabbed or touched by this conspecific
Out of arm’s reach	Individual is too far away from a conspecific to grab or touch it and could not be grabbed or touched by this conspecific
Out of sight	Individual is not visible to the observer and no other behavior could be concluded when taking other conspecifics into account (e.g. out of arm’s reach to everyone)

### Statistics

To test for significant differences between two frequencies (success vs. failure, comparison of performances between two individuals, and comparison of the two distance-categories “within arm’s reach to another conspecific” and “in close contact to another conspecific” for individual gibbons), the non-parametric Chi-square-test was applied (Geissmann 2002; Siegel and Castellan Jr. 1988). In all Chi-square tests, the degree of freedom (*df*) was 1.

In order to test for significant differences between three frequencies (comparison of performances between three individuals), the non-parametric Kruskal–Wallis-test was applied (Kruskal and Wallis 1952). Subsequently, a *post-hoc* test with pairwise comparisons and Bonferroni correction was conducted (Armstrong 2014). Both tests were performed using *IBM SPSS (Statistical Package for Social Science) version 25* for Windows.

To determine if a positive success rate was established, the ratio of correctly solved trials and failed trials over time was analyzed. To examine whether there was a significant transition in the gibbons’ performances regarding the success rate, a Spearman Rank Correlation test was conducted. Since the number of trials per training session was determined by each individual’s participation (i.e., motivation), there was no consistent number of trials per training session. In order to obtain a more reliable outcome, only sessions that contained at least 10 trials were included in the analysis (i.e., a reduced data set).

Spearman rank correlation tests (Siegel and Castellan Jr. 1988) were computed using *StatView 5.0.1* software on an *iMac PowerMac 4.2* (for success rates) and *IBM SPSS version 25* for Windows (for progression rates). Correlation coefficients Rho (in absolute values) were interpreted according to Taylor (1990): r_s_ ≤ 0.35 (weak correlation); 0.36 ≤ r_s_ ≤ 0.67 (moderate correlation); 0.68 ≤ r_s_ ≤ 1 (strong correlation).

A *p*-value ≤ 0.05 was considered as statistically significant.

## Results

### Phase 1: first training phase

The results and respective statistical values for the first training phase for all individuals are shown in Table 3. Overall, three out of four gibbons solved the rope pulling task significantly more often than they failed it. This meant that they could proceed with the second training phase.

**Table 3:** Results of the first training phase

Individual	Number of received training sessions	Number of successful trials/total number of trials	Individual accepted to Training phase 2: yes/no	Progression of the mean time (until success) per session over time	Interpretation^1^ of correlation coefficient Rho	Ratio successful vs. failed trials over time (i.e., success rate) with reduced data set (at least 10 trials)	Interpretation^1^ of correlation coefficient Rho with reduced data set (at least 10 trials)
Lelle	19	138/151; (*χ*^2^ = 103.48, *n* = 151, ***p* < 0.001**)	yes	Rho = 0.127, *n* = 18, *p* = 0.616	Weak positive trend	Rho = − 0.387, n = 8, *p* = 0.2556	Moderately negative trend
Elly	NA	NA	no	NA	NA	NA	NA
Elliot	51	253/271; (*χ*^2^ = 203.78, *n* = 271, ***p* < 0.001**)	yes	Rho = − 0.554, *n* = 44, ***p* < 0.01**	Moderately negative trend	Rho = 0.434, *n* = 11, *p* = 0.2215	Moderately positive trend
Edith	21	190/202; (*χ*^2^ = 156.85, *n* = 202, ***p* < 0.001**)	yes	Rho = − 0.306, *n* = 18, *p* = 0.217	Weak negative trend	Rho = 0.269, *n* = 14, *p* = 0.4821	Weak positive trend

^1^Terminology afterTaylor (1990)

A *p*-value ≤ 0.05 was considered as statistically significant

To assess whether the animals’ performances improved over time, the progression of the mean time until success per session was analyzed. Lelle needed more time to solve the task in the end of the first training phase compared to the beginning. Both, Elliot and Edith were faster in solving the task in the end of this training phase than they were in the beginning. To determine if positive success rates could be issued for each individual, the ratio between correctly solved and failed trials over time was analyzed, taking the reduced data set (see above) into account. Lelle solved less trials correctly over time, whereas both Elliot and Edith solved increasingly more trials correctly over time.

### Phase 2: second training phase

The results and respective statistical values for the second training phase for all individuals are shown in Table 4. No individual was significantly more often successful in the required task than failing it. Looking at the progression of the mean times until success per session over time, Elliot and Edith both developed a positive trend, meaning that both individuals became faster in solving the task correctly over time. When analyzing the success rates for both individuals, strong positive trends were found. This shows that both individuals solved more trials correctly by the end of the second training phase than in the beginning. These results were significant (Table 4). Edith was the only one for which the distance between the two rope ends could be increased. In several steps, the initial distance of 11 cm could be enlarged to a final distance of 62 cm.

**Table 4:** Results for the second training phase

Individual	Number of received training sessions	Number of successful trials/total number of trials	Individual accepted to Test phase: yes/no	Progression of the mean time (until success) per session over time	Interpretation^1^ of correlation coefficient Rho	Ratio successful vs. failed trials over time (i.e., success rate) with reduced data set (at least 10 trials)	Interpretation^1^ of correlation coefficient Rho with reduced data set (at least 10 trials)
Lelle	63	0/157	no	NA	NA	NA	NA
Elliot	47	19/140; (*χ*^2^ = 74.31, *n* = 140, ***p* < 0.001**)	no	Rho = 0.434, *n* = 12, *p* = 0.159	Moderately positive trend	Rho = 0.762, *n* = 30, *p* < 0.0001*	Strong positive trend*
Edith	97	105/333; (*χ*^2^ = 45.43, *n* = 333, ***p* < 0.001**)	no	Rho = 0.071, *n* = 54, *p* = 0.608	Weak positive trend	Rho = 0.881, *n* = 8, ***p* = 0.0201**	Strong positive trend

The asterisks in Elliot’s row indicate an unreduced data set (there were no sessions that featured at least 10 trials)

^1^Terminology afterTaylor (1990)

A *p*-value ≤ 0.05 was considered as statistically significant

Due to time limits, the training phase could not have been prolonged. Usually with this project design, the results of the training phases would stipulate to terminate the study. But since this project was of experimental nature, the test apparatus was installed nevertheless, to explore what the gibbons would make out of this situation. And even though it remains unclear whether Elliot and Edith conceived the task entirely, they did exhibit an increasing success rate.

### Phase 3: test phase

In the test phase, Lelle pulled a rope 24 times in total. Elliot showed a little more interest in the ropes with 40 pulls in total. Edith exhibited most interest in the ropes and pulled them 77 times in total.

No cooperative behavior was recorded. On four occasions two individuals were sitting together in front of the apparatus during the test phase. Each time, one gibbon was actively pulling one rope, while the other one was present, but not pulling the second rope. Edith was the possible cooperation partner in all four occasions, with Lelle and Elliot being the counterpart of a possible cooperation dyad twice.

### Behavioral observations

#### Distances between the animals

**Fig. 2:**
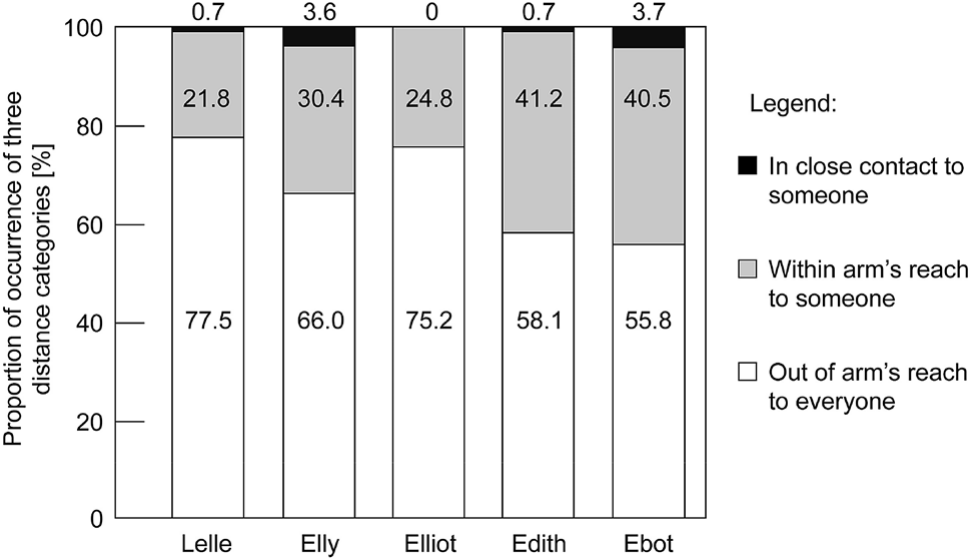
Proportion of occurrences of the three distance categories for each of the five family group members. Definitions for the distance categories are listed in Table 2

Behavioral observations revealed that all gibbons spent most of their time in the widest distance class: “out of arm’s reach to everyone” (Fig. 2). Each individual used this distance class more often than the other two distance classes combined: “Within arm’s reach to another conspecific” and “in close contact to another conspecific”. This difference was significant (*p* < 0.001) for every individual (Lelle: *χ*^2^ = 1.16 × 10^10^, *n* = 3862; Elly: *χ*^2^ = 360.81, *n* = 3514; Elliot: *χ*^2^ = 944.31, *n* = 3715; Edith: *χ*^2^ = 100.42, *n* = 3791; Ebbot: *χ*^2^ = 47,41, *n* = 3546).

#### Social behavior

During observations, social grooming behavior for each individual was recorded. The data showed that most of the grooming was displayed by Edith. She performed this behavior in 13.24% of the observed time. Elly performed grooming 4.78% of the observed time. Elliot, Ebbot, and Lelle displayed grooming only rarely, namely in 1.8%, 0.12%, and 0.05% of the observed time, respectively.

Most of the observed play behavior occurred between Edith and Ebbot (on average 2.4 times per hour). Edith and Elliot played together on average 1.2 times per hour. Between Elliot and Ebbot, play behavior was recorded on average 0.6 times per hour. Lelle and Elly both played with their offspring only twice during the whole study.

Conflict behavior was almost never seen. During the whole study, it occurred only once between Lelle and Edith, and once between Elly and Ebbot.

## Discussion

In order to discover whether or not cooperative behaviors occur among gibbons, the white-handed gibbons at Kolmården Wildlife Park in Sweden were presented with an experimental problem-solving task, in which two individuals were required to simultaneously pull a rope in order to receive a food reward.

In the first training phase, three individuals showed that they were able to pull a rope in order to obtain a food reward. Furthermore, after having learned this task, they required only a few seconds to complete the task. Both findings go along with those of Beck (1967), whose gibbons were also able to solve all presented rope-pulling tasks. Due to the lack of time, however, no control condition (e.g., available rope without food reward) could be included in the present study. Such a control should be considered for future examinations to further investigate the gibbons’ extent of insight. Beck (1967) reported that his gibbons repeatedly pulled the rope even though no food reward was connected to it. This raises doubt that the individuals actually understood the physical properties of the task and may indicate that rope-pulling was merely a conditioned behavior.

The second training phase, in which the individuals were required to learn to pull two ropes simultaneously to get the reward, already suggested cognitive limitations. After having learned to pull one rope it proved difficult to expand the learned behavior. Adult male Lelle never managed to manipulate two rope ends at the same time, even though he seemed motivated to engage in the training. A reason for his limited cognitive flexibility may have been his advanced age (30 years). It is presumed that wild gibbons have a longevity of 25 to 35 years, although in captivity an age of up to 60 years has been reported (Geissmann et al. 2009). Either way, Lelle’s age may have impaired his learning capability, which could have resulted in a delayed learning process (Geinisman et al. 1995). In contrast, both the juvenile female Edith and the subadult female Elliot exhibited the onset of the required behavior. At the end of the second training phase, Elliot pulled the two rope ends together progressively more frequently and more confidently. Edith’s training progress was even more advanced and the distance between the two rope ends could progressively be increased.

During the actual test phase, no cooperative behaviors were displayed, and thus, these gibbons were not able to succeed in the problem-solving task. The motivation level of all the gibbons declined drastically after they discovered that single-rope-pulling behavior would no longer lead to success. They were more likely to lose interest and ignore the apparatus instead of persistently trying to achieve the reward, one way or another. This goes along with the findings of previous studies which have reported low levels of motivation in gibbons during cognitive challenges and emphasized that low motivation, rather than the absence of cognitive abilities, may have restrained their success (Cunningham 2006; Martinez Sierra 2013). Yamamoto (2021) also highlights the importance of considering the absence of motivation when interpreting the absence of a certain behavior.

Furthermore, the testing apparatus in this study might have been too complex or unintuitive, and the interplay of the two ropes may not have been obvious enough for the gibbons to grasp the concept. Mendres and de Waal (2000) discussed the phenomenon of the absence of cooperative behaviors due to an unclear experimental design instead of missing cognitive abilities. On the other hand, D’Agostino and Cunningham (2015) concluded in their study that gibbons are most likely not able to use previously learned information in order to complete a subsequent task, which is based on that gained information.

It remains unclear if the lack of motivation or actual cognitive limitations were the reason for the absence of cooperative behaviors in this gibbon group. This subject clearly needs more attention to provide a more solid ground for assumptions regarding cooperative behaviors within gibbons.

In order to approach the origin and evolution of cooperative behaviors within the primate taxa, not only performances of apes (Hominoidea) have to be taken into account but those of monkey species as well. Several species of Old World monkeys (Cercopithecoidea)–the sister group of the apes–have been examined regarding their abilities to solve a given task in cooperation with their conspecifics. Many of the tested monkey species were not able to develop cooperative problem-solving skills, including the sooty mangabey (Warden and Galt 1943), Guinea baboon (Fady 1972), rhesus macaque (Petit et al. 1992; Warden and Galt 1943), and Tonkean macaque (Petit et al. 1992). However, wild Barbary macaques (Molesti and Majolo 2016), long-tailed macaques (Stocker et al. 2020), and Japanese macaques (Kaigaishi et al. 2019) have been shown to be successful during respective cooperation tasks.

Several studies have also reported occurrences of cooperative behaviors in various species of the New World monkeys (Platyrrhini), including cotton-top tamarins (Cronin et al. 2005; Cronin and Snowdon 2008), common marmosets (Werdenich and Huber 2002), and brown capuchins (Brosnan et al. 2006; de Waal and Davis 2003; Hattori et al. 2005; Mendres and de Waal 2000).

We propose that the occurrence of cooperative behaviors in the two lineages, the Catarrhini and the Platyrrhini might be best explained as a result of convergent evolution. The gap between families that do exhibit the corresponding behaviors seems to be too large to consider this trait to be a homology.

It appears to be more likely that similar selective pressures and cognitive adaptations have independently caused the development of cooperative behaviors.

Lots of research has already been devoted to understanding the underlying processes and necessary cognitive requirements and abilities that enable cooperation between individuals. Yet, there is no common theory why, how, and when cooperative behaviors have evolved. It is reasonable to assume, however, that somewhat sophisticated socio-cognitive competencies are driving factors.

It has been hypothesized that cognitive abilities are constrained by the complexity of the animals’ social life (Dunbar 1998; Humphrey 1976). Furthermore, Burkart et al. (2014) suggest that allomaternal care is the best index for proactive prosocial behaviors, which are thought to be one of the factors that foster cooperative behaviors. The gibbons in their study showed low proactive prosocial behaviors (Burkart et al. 2014).

Gibbons typically live in small stable family groups (Bartlett 2011; Chivers 1977; Leighton 1987) with few conspecifics to interact with, and they spend comparatively little time with social interactions compared to primates living in larger groups. Bartlett (2003) reported a relatively low proportion of social interactions for the white-handed gibbon during field observations (annual mean of 11.3%). This tendency was reflected in the behavioral observations in this study. Black crested gibbons (*Nomascus concolor*) have also been observed to devote the lowest proportion of their time to social interactions *vs*. other activities (Sheeran 1993).

Due to the lack of research on these apes, it is too early and impossible to speculate how gibbon cognition fits into this puzzle. It is clear that more research in this area is desperately needed. The present and mentioned studies suggest, it may require more time and effort to thoroughly conduct cognitive experiments within this primate family, which is something that should be taken into account before planning any study that requires gibbons’ participation.

For future experiments on cooperative behaviors, however, it would also be interesting to include the siamang (*Symphalangus syndactulus*). Siamangs live sympatrically with white-handed gibbons and agile gibbons (*H. agilis*) (Geissmann 2003). Yet, siamangs differ from these gibbon species in several aspects of their social organization. For instance, siamang fathers are often involved in allomaternal care by carrying infants during their second year of life (Alberts 1987; Chivers 1974; Lappan 2008). Additionally, siamangs appear to have more cohesive groups than white-handed gibbons and pileated gibbons (*H. pileatus*) (Chivers 1976; Geissmann et al. 2020; Palombit 1996), and may even have a stronger pair-bond than other gibbon species (Geissmann et al. 2020). Munir (2018) reported siamangs’ success and high potential in cognitive tests. It would thus be worthwhile to investigate whether siamangs are more successful in cooperative problem-solving tasks than the white-handed gibbons in this study.

## Supplementary Information

Below is the link to the electronic supplementary material.

Supplementary file1 (MP4 2720 KB)

## Data Availability

For accessing the data collected in this study, please contact the corresponding author. Video files cannot be shared due to protection of animal keepers’ privacy
