# Correction: A cooperation experiment with white-handed gibbons (*Hylobates lar*)

**DOI:** 10.1007/s10329-023-01078-5

**Published:** 2023-07-14

**Authors:** Nora T. Kopsch, Thomas Geissmann

**Affiliations:** 1 Department of Physics, Chemistry and Biology, Linköping University, Linköping, Sweden; 2 Anthropological Department, University Zurich-Irchel, Zurich, Switzerland; 3 Department of Philosophy, Cognitive Science, Lund University, Lund, Sweden

**Correction: Primates** 10.1007/s10329-023-01068-7

In the original publication of the article, the corresponding author’s affiliation should be changed to “Department of Philosophy, Cognitive Science, Lund University, Lund, Sweden” instead of “Behavioural Ecology Research Group, Anglia Ruskin University, Cambridge, UK” and the email address should be nora_tabea.kopsch@lucs.lu.se instead of norko743@student.liu.se due to an update in the funding note which is updated in the original article.

